# Survival Outcomes in Older Adult Acute Lymphoblastic Leukemia Patients Analyzed by Facility Volume and Type: A National Cancer Database Analysis

**DOI:** 10.1002/cnr2.2162

**Published:** 2024-08-08

**Authors:** Kaitlyn C. Dykes, Jiling Chou, Allison O. Taylor, Albert C. Shu, Sarah E. Mudra, Xiaoyang Ma, Jaeil Ahn, Catherine E. Lai

**Affiliations:** ^1^ Department of Medicine MedStar Georgetown University Hospital Washington DC USA; ^2^ Department of Medicine, Division of Hematology and Oncology University of California San Diego Moores Cancer Center San Diego California USA; ^3^ Department of Biostatistics, Bioinformatics and Biomathematics Georgetown University Washington DC USA; ^4^ Department of Medicine, Division of Hematologic Malignancies & Cellular Therapy Duke Cancer Institute Durham North Carolina USA; ^5^ Lombardi Comprehensive Cancer Center and MedStar Georgetown University Hospital Washington DC USA; ^6^ Perelman‐University of Pennsylvania Hospital Philadelphia Pennsylvania USA

**Keywords:** epidemiology, hematalogical cancer, leukemia, survival

## Abstract

**Bakground:**

It is important to understand the outcomes of adult acute lymphoblastic leukemia (ALL) patients at different facilities as treatment paradigms change.

**Aims:**

Our primary objective was to determine adult ALL overall survival (OS) by facility volume and type. Secondary objectives included identifying sociodemographic factors that may have impacted outcomes and analyzing treatment patterns by facility volume and type.

**Methods:**

This was a retrospective analysis of the National Cancer Database (NCDB) that included patients ≥40 years diagnosed with ALL between 2004 and 2016.

**Results:**

A total of 14 593 patients were included in this study. Univariate OS was greatest at low volume (LV) and community programs (CPs) and the least at high volume (HV) and academic programs (AP). This difference was lost after multivariable Cox proportional hazards model analysis, which found no difference in survival by facility volume or type, however, survival was significantly influenced by age, race, Hispanic ethnicity, insurance, and residence location (*p* < 0.05). Patients treated at HV and APs compared to LV and CP received more anti‐neoplastic directed therapy.

**Conclusion:**

Our results suggest treatment facility volume and type do not impact older adult ALL patient (≥40 years) survival, however confounding sociodemographic differences do impact survival outcomes, despite more aggressive and novel treatment approaches provided at HV and APs.

## 1 Introduction

Acute lymphoblastic leukemia (ALL) is a heterogeneous group of hematologic malignancies (HMs) that arise from B or T lymphoid progenitor cells [1, 2]. Today, most ALL deaths occur in adults [2, 3]. ALL incidence occurs in a bi‐modal frequency with a second peak in older adulthood, given a rising incidence starting in the fourth decade of life [3]. Outcomes in adult ALL are complex. With increased age, patients often acquire other comorbid conditions and may be less fit compared to younger patients making treatment more challenging in some cases, however underlying disease biology starting around 40 years of age has also been shown to be more aggressive compared to younger patients [1, 4, 5, 6].

In recent decades, outcomes in adult ALL have dramatically improved. Analysis of the Surveillance, Epidemiology, and End Results (SEER) database from 1980 to 2017 has shown that over the past 40 years, 5‐year survival in patients 40–59 years has improved from 14% to 43%, and in patients 60–69 years from 10% to 29% [7]. Those in the oldest cohort, ≥70 years, continued to have the poorest outcomes, yet 5‐year survival still improved from 3% in the 1980s to 13% in 2010–2017.

Reasons for outcome improvement are multifactorial, including risk stratification by minimal residual disease, utilization of pediatric‐based chemotherapy regimens in combination with risk‐adapted stem cell transplantation (SCT) in select adults, tyrosine kinase inhibitor (TKI) use in patients with Philadelphia (Ph) chromosome‐positive ALL, and the advent of immunotherapy‐based treatment options that are better tolerated even in older adults [8, 9, 10, 11, 12, 13, 14, 15, 16]. Treatment of adult ALL patients of all ages remains an area of ongoing investigation, with continued outcome improvements being made even in older patients [13].

The purpose of our study was to determine the impact of treatment facilities on outcomes in older adults with ALL. Our primary objective was to determine overall survival (OS) by facility volume and type. Secondary objectives included identifying sociodemographic factors that may have impacted patient outcomes and analyzing treatment patterns by facility volume and type.

## 2 Methods

### 2.1 Data Source and Study Population

This study was a retrospective, cross‐sectional, Institutional Review Board‐exempt analysis of the 2004–2016 National Cancer Database (NCDB). The database is sourced by more than 15 000 Commission on Cancer‐accredited facilities and is estimated to represent more than 72% of newly diagnosed cancer cases nationwide [17]. Patients ≥40 years of age newly diagnosed with ALL and who received all or some treatment at the reporting facility were included. Patients were excluded if reporting facility volume or facility type was missing. Facility volume was classified as high volume (HV) and low volume (LV), where HV was defined as the top 9 percentile (>91%), similar to prior NCDB studies [18, 19]. Facility type was described as academic programs (AP) and community programs (CPs). APs included academic/research center cancer programs and integrated network programs, which were post‐graduate training sites and/or a group of facilities that offered integrated cancer care services as defined by the NCDB. CPs included CPs and comprehensive CPs that cared for at least 100 new cancer cases annually. Because of NCDB policy to maintain patient privacy, facility type was not available for patients <40 years of age, thus patients <40 were excluded.

### 2.2 Statistical Analysis

Baseline characteristics were abstracted by facility volume and type. Summary statistics included frequency (%) for categorical variables and median [IQR] for continuous variables. Group differences were compared with Chi‐squared or Kruskal–Wallis rank sum tests. As defined by NCDB Participant User Data File, sociodemographic covariates included: age, sex, race, ethnicity, insurance status, Charlson Comorbidity Index (CCI), median annual income, education, population density per census classifications of urban, metro, and rural, and distance from clinic. To define OS and time to treatment (TTT), time was considered as last “contact or death, months from diagnosis,” and event was considered as “vital status last contact or death or last observed treatment date.” Kaplan–Meier (KM) curves were generated for OS and TTT for facility volume and type, stratified by age. The KM method was used to estimate median OS and the log rank test was used to compare OS across predictor variables. Multivariable Cox proportional hazards models regarding OS were used to evaluate the covariates associated with OS, stratified by year of diagnosis and age at diagnosis ≥40 years. Odds ratios (OR) with 95% confidence intervals (CI) were calculated and depicted as a forest plot for treatments offered by facility volume and type. Treatment was classified as any chemotherapy, immunotherapy, radiation, SCT, and palliative care. Analysis was conducted with R software 4.0.3.

## 3 Results

### 3.1 Descriptive Statistics

A total of 14 593 were ≥40 years of age and were included in the study. Overall 9708 (66.5%) of patients were treated at APs and 4885 (33.5%) patients were treated at CPs (Table 1). Overall 7589 (52.0%) of patients were treated at HV centers and 7004 (48.0%) at LV centers. Amongst the ≥40 cohort most APs were also HV (71.4%), however, there was a proportion of LV APs (28.6%) (Table 1). Most CPs were also LV centers (86.5%). Statistically different baseline characteristics between patients treated at HV versus LV facilities included facility type, age at diagnosis, ethnicity, insurance, CCI, median income, education, urban versus rural, and distance from clinic (Table 1). Statistically different baseline characteristic variables between the patients treated at CP versus AP included center volume, age at diagnosis, race, insurance, CCI, median income, urban versus rural, and distance from clinic.

**TABLE 1:** Baseline characteristics for patients ≥40 years, stratified by center volume and facility type.

			Center volume (%)			Facility type (%)		
		Overall	Low ≤91%	High >91%	*p*	Community	Academic	*p*
*n*		14 593	7004	7589		4885	9708	
Facility type (%)	Community program	4885 (33.5)	4227 (60.4)	658 (8.7)	**<0.001**			
	Academic program	9708 (66.5)	2777 (39.6)	6931 (91.3)				
Center volume (%)	Low volume ≤91%	7004 (48.0)				4227 (86.5)	2777 (28.6)	**<0.001**
	High volume >91%	7589 (52.0)				658 (13.5)	6931 (71.4)	
Age at diagnosis (median [IQR])		60.0 [50.0, 70.0]	62.0 [52.0, 73.0]	58.0 [49.0, 67.0]	<0.001*	62.0 [52.0, 73.0]	59.0 [50.0, 68.0]	<0.001*
Sex (%)	Male	7646 (52.4)	3664 (52.3)	3982 (52.5)	0.862	2557 (52.3)	5089 (52.4)	0.944
	Female	6947 (47.6)	3340 (47.7)	3607 (47.5)		2328 (47.7)	4619 (47.6)	
Race (%)	White	12 526 (86.8)	6030 (87.0)	6496 (86.6)	0.887	4336 (89.5)	8190 (85.4)	**<0.001**
	Black	1171 (8.1)	553 (8.0)	618 (8.2)		279 (5.8)	892 (9.3)	
	Asian	457 (3.2)	220 (3.2)	237 (3.2)		147 (3.0)	310 (3.2)	
	Other	284 (2.0)	132 (1.9)	152 (2.0)		83 (1.7)	201 (2.1)	
Ethnicity (%)	NonSpanish/NonHispanic	11 954 (85.8)	5815 (88.6)	6139 (83.3)	**<0.001**	4008 (86.6)	7946 (85.4)	0.057
	Spanish/Hispanic	1976 (14.2)	745 (11.4)	1231 (16.7)		619 (13.4)	1357 (14.6)	
Insurance (%)	Uninsured	682 (4.9)	325 (4.7)	357 (5.0)	**<0.001**	238 (5.0)	444 (4.8)	**<0.001**
	Private	6459 (46.0)	2886 (42.0)	3573 (49.7)		2046 (42.8)	4413 (47.6)	
	Medicaid	1355 (9.6)	548 (8.0)	807 (11.2)		330 (6.9)	1025 (11.1)	
	Medicare	5375 (38.3)	3032 (44.2)	2343 (32.6)		2116 (44.2)	3259 (35.2)	
	Other	176 (1.3)	73 (1.1)	103 (1.4)		52 (1.1)	124 (1.3)	
Charlson Comorbidity Index (%)	0	10 437 (71.5)	4825 (68.9)	5612 (73.9)	**<0.001**	3385 (69.3)	7052 (72.6)	**<0.001**
	1 or 2	3731 (25.6)	1949 (27.8)	1782 (23.5)		1347 (27.6)	2384 (24.6)	
	3	425 (2.9)	230 (3.3)	195 (2.6)		153 (3.1)	272 (2.8)	
Median income (%)	<$38 000	2548 (17.5)	1209 (17.4)	1339 (17.7)	**<0.001**	854 (17.6)	1694 (17.5)	**<0.001**
	38 000–47 999	3261 (22.5)	1585 (22.7)	1676 (22.2)		1173 (24.1)	2088 (21.6)	
	48 000–62 999	4004 (27.6)	2016 (28.9)	1988 (26.3)		1435 (29.5)	2569 (26.6)	
	≥63 000	4711 (32.4)	2158 (31.0)	2553 (33.8)		1400 (28.8)	3311 (34.3)	
Education; no HS degree (%)	≥21.0%	2961 (20.4)	1308 (18.8)	1653 (21.9)	**<0.001**	1007 (20.7)	1954 (20.2)	0.741
	13.0%–20.9%	3558 (24.5)	1725 (24.7)	1833 (24.3)		1199 (24.6)	2359 (24.4)	
	7.0%–12.9%	4590 (31.6)	2283 (32.7)	2307 (30.5)		1539 (31.6)	3051 (31.6)	
	<7.0%	3425 (23.6)	1660 (23.8)	1765 (23.4)		1122 (23.1)	2303 (23.8)	
Urban/rural (%)	Metro	12 032 (82.5)	5698 (81.4)	6334 (83.5)	**0.003**	3826 (78.3)	8206 (84.5)	**<0.001**
	Rural	642 (4.4)	336 (4.8)	306 (4.0)		268 (5.5)	374 (3.9)	
	Urban	1919 (13.2)	970 (13.8)	949 (12.5)		791 (16.2)	1128 (11.6)	
Distance from clinic (%)	>100 miles	1347 (9.3)	217 (3.1)	1130 (14.9)	**<0.001**	218 (4.5)	1129 (11.7)	**<0.001**
	10–100 miles	7430 (51.1)	2871 (41.1)	4559 (60.3)		2101 (43.2)	5329 (55.1)	
	10 miles or less	5763 (39.6)	3891 (55.8)	1872 (24.8)		2550 (52.4)	3213 (33.2)	

*Note: p*‐values were calculated from Chi‐square test with Yate's correction, unless marked with an asterix (*), which were calculated from Kruskal–Wallis rank sum test. *p*‐values <0.05 were considered significant and were bolded.

### 3.2 Overall Survival

**FIGURE 1:**
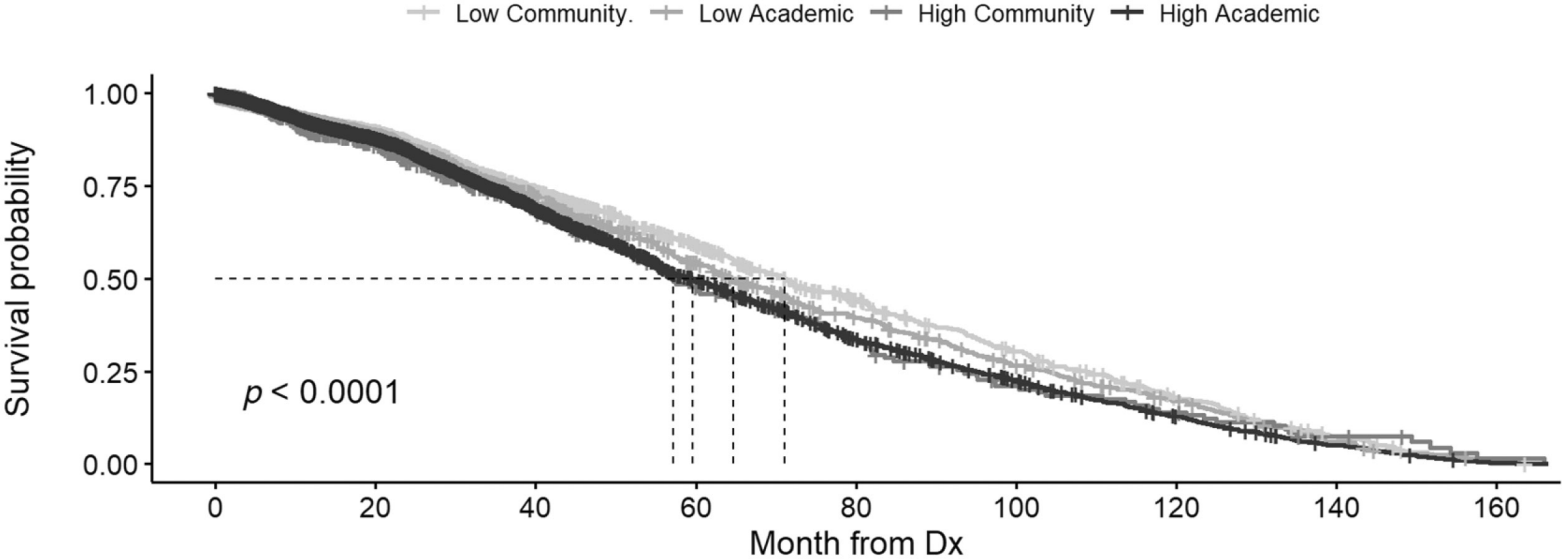
Kaplan–Meier (KM) overall survival (OS) curve including patients ≥40 years, by facility volume and type. OS was censored by death or last follow‐up. Stratified analysis of patients ≥40 years found OS was longest at LV and CP (71.1 months) and the shortest at HV and AP (59.6 months), *p* < 0.001. A total of 3429 observations were excluded from the analyses due to missing data.

**FIGURE 2:**
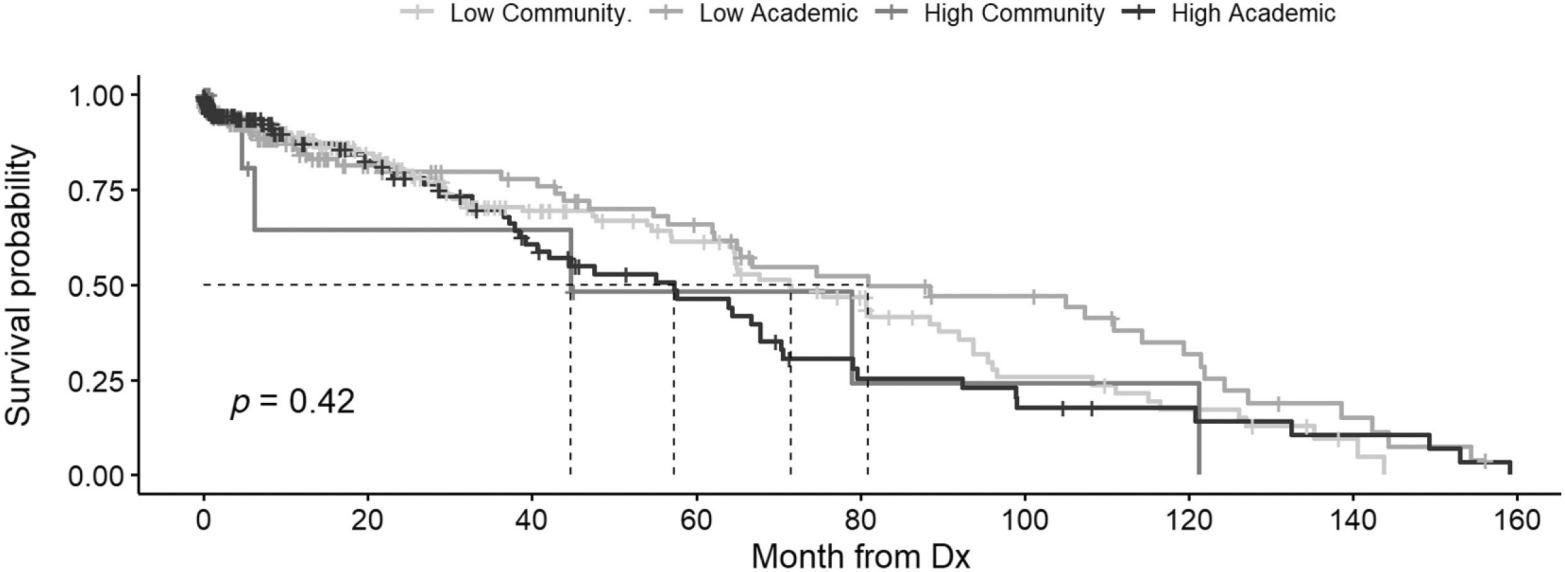
Kaplan–Meier (KM) overall survival (OS) curve including patients ≥40 years who received no treatment other than palliative care (no immunotherapy, chemotherapy, radiation, and transplant) by facility volume and type. OS was censored by death or last follow‐up.

KM curves for OS by facility volume and type were analyzed. Univariate analysis of patients ≥40 years found OS was greater at LV compared to HV centers with mOS 68.7 mo (95% CI 65.2–71.5) versus mOS 59.5 mo (95% CI 56.6–62.3, *p* < 0.001) (Table S1). Univariate analysis found OS was greater at CPs compared to APs with mOS 69.2 mo (95% CI 65.0–72.6) versus mOS 61.2 mo (95% CI 58.5–63.4, *p* < 0.001) (Table S1). Stratified KM analysis found OS was greatest at LV and CP and the least at HV and AP, mOS 71.1 mo (95% CI 66.6–75.8) versus mOS 59.6 mo (95% CI 56.6–62.7, *p* < 0.001) (Figure 1). KM OS analysis of patients who received no treatment other than palliative care by facility volume and type found OS between groups was not different (*p* = 0.42) (Figure 2).

Multivariable Cox proportional hazards model analysis between HV versus LV facilities found a hazard ratio (HR) 1.08 (95% CI 0.99–1.17, *p* = 0.096) and between APs versus CPs HR 1.01 (95% CI 0.92–1.11, *p* = 0.81) (Table 2). Variables associated with a significantly increased HR included: increased age (HR 1.01, 95% CI 1.01–1.01, *p* < 0.001), other/Asian versus white (HR 1.33, 95% CI 1.07–1.66, *p* = 0.012; HR 1.22, 95% CI 1.04–1.44, *p* = 0.015) and Spanish/Hispanic ethnicity versus nonSpanish/Hispanic ethnicity (HR 1.23, 95% CI 1.1–1.37, *p* < 0.001). Variables with a significantly decreased HR included: private/medicare insurance versus no insurance (HR 0.73, 95% CI 0.63–0.85, *p* < 0.001; HR 0.65, 95% CI 0.54–0.77, *p* < 0.001) urban/rural versus metro (HR 0.83, 95% CI 0.74–0.93, *p* = 0.002; HR 0.81, 95% CI 0.68–0.98, *p* = 0.002), and distance from clinic 10 to 100/<10 miles versus >100 miles (HR 0.87, 95% CI 0.77–0.98, *p* = 0.019; HR 0.8, 95% CI 0.7–0.91, *p* = 0.001).

**TABLE 2:** Multivariable Cox proportional hazards model results regarding overall survival, stratified by year of diagnosis and age at diagnosis ≥40 years.

Variable	HR (95%CI)	*p*
Academic vs. community program	1.01 (0.92, 1.11)	0.81
High vs. low volume (91% cut point)	1.08 (0.99, 1.17)	0.096
Age	1.01 (1.01, 1.01)	**<0.001**
Sex (female vs. male)	1.03 (0.97, 1.11)	0.329
Black vs. White	1 (0.87, 1.14)	0.951
Asian vs. White	1.22 (1.04, 1.44)	**0.015**
Other vs. White	1.33 (1.07, 1.66)	**0.012**
Ethnicity Spanish/Hispanic vs. nonSpanish/Hispanic	1.23 (1.1, 1.37)	**<0.001**
Private insurance vs. none	0.73 (0.63, 0.85)	**<0.001**
Medicaid vs. none	0.89 (0.75, 1.05)	0.168
Medicare vs. none	0.65 (0.54, 0.77)	**<0.001**
Other insurance vs. none	1.22 (0.9, 1.65)	0.21
Charlson 1–2 vs. 0	1.05 (0.96, 1.14)	0.316
Charlson 3 vs. 0	1.14 (0.84, 1.55)	0.409
Rural vs. metro	0.81 (0.68, 0.98)	**0.03**
Urban vs. metro	0.83 (0.74, 0.93)	**0.002**
Median income		
< $38 000 vs. 38 000–47 999	1.05 (0.93, 1.18)	0.467
< $38 000 vs. 48 000–62 999	1.12 (0.99, 1.28)	0.074
< $38 000 vs. ≥ 63 000	1.15 (1, 1.33)	0.056
Distance from clinic		
10–100 miles vs. >100 miles	0.87 (0.77, 0.98)	**0.019**
10 miles or less vs. >100 miles	0.8 (0.7, 0.91)	**0.001**

*Note:* Time was defined as last “contact or death, months from diagnosis,” and event was defined as “vital status last contact or death” (1 = death). *p*‐values <0.05 were considered significant and were bolded.

Abbreviation: HR, hazard ratio.

### 3.3 Treatment

**FIGURE 3:**
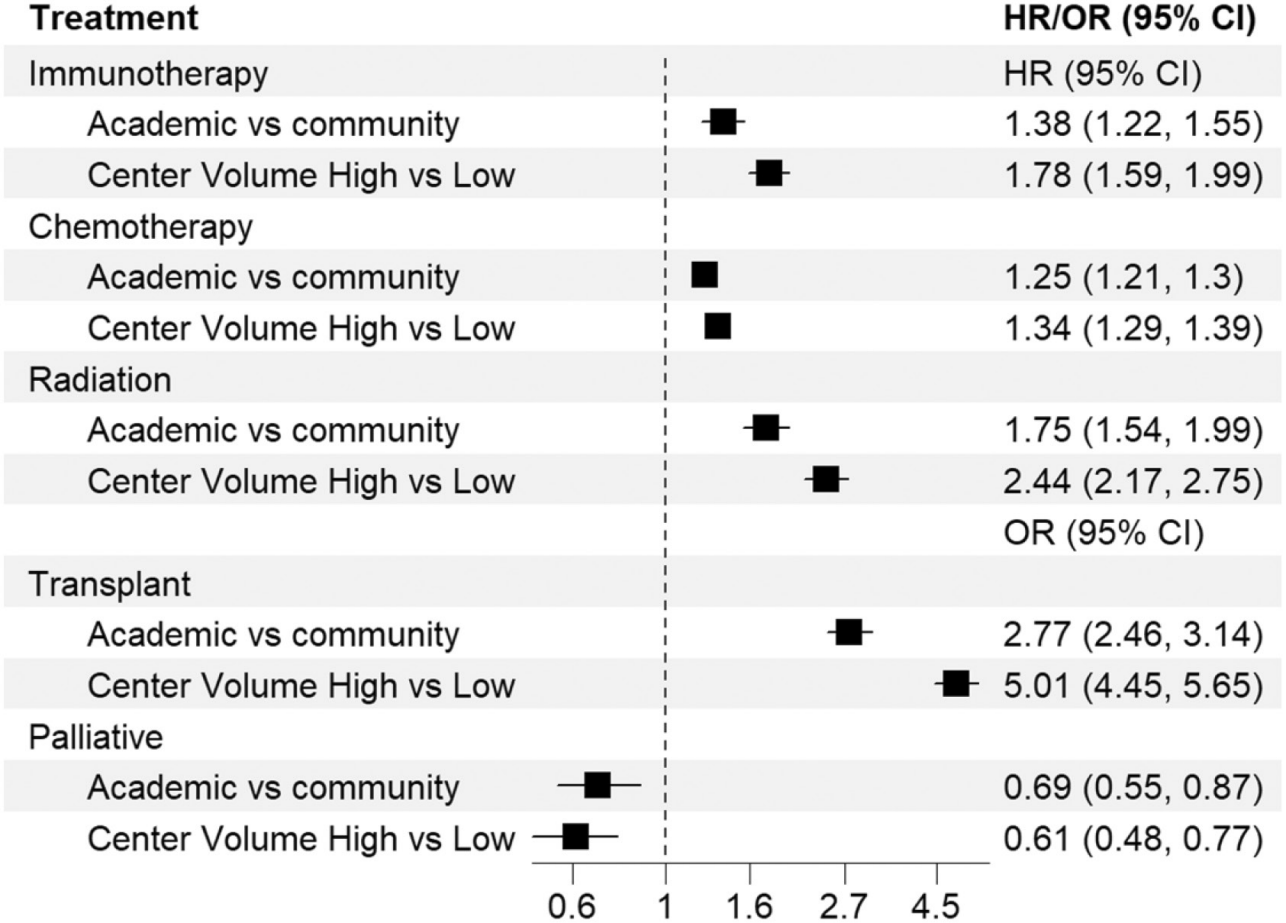
Hazard ratio (HR) and odds ratio (OR) by initial treatment received (immunotherapy, chemotherapy, radiation, transplant, and palliative) by facility volume and type for patients ≥40.

Multivariable Cox analysis found TTT was greatest at LV and CP and the shortest at HV and CP (mTTT 7 days, 95% CI 6–7 versus mTTT 4 days, 95% CI 3–4; *p* < 0.001) (Table S2). Amongst patients ≥40, 9.5% received immunotherapy, 90.5% chemotherapy, 10.0% radiation, and 13.8% underwent transplant (Table S3). Patients treated at HV versus LV were more likely to receive immunotherapy (HR 1.78, 95% CI 1.59–1.99, *p* < 0.001), chemotherapy (HR 1.34, 95% CI 1.29–1.39, *p* < 0.001), and radiation (HR 2.44, 95% CI 2.17–2.75, *p* < 0.001). They also had greater likelihood of undergoing transplant (OR 5.01, 95% CI 4.45–5.65, *p* < 0.001). Patients treated at APs versus CPs were more likely to receive immunotherapy (HR 1.38, 95% CI 1.22–1.55, *p* < 0.001), chemotherapy (HR 1.25, 95% CI 1.21–1.3, *p* < 0.001), radiation (HR 1.75, 95% CI 1.54–1.99, *p* < 0.001), and had a greater likelihood of undergoing transplant (OR 2.77, 95% CI 2.46–3.14, *p* < 0.001) (Figure 3). HV versus LV and AP versus CP both had a lower likelihood of providing palliative treatment, respectively, OR 0.61 (95% CI 0.48–0.77, *p* < 0.001) and OR 0.69 (95% CI 0.55–0.87, *p* = 0.002).

## 4 Discussion

### 4.1 Survival Outcomes

The impact of treatment by facility volume and type in older adult ALL is nuanced in this large retrospective NCDB study of adults with ALL. Herrin, we highlight important findings. Univariate analysis found significant inferior survival at HV and APs compared to LV and CPs amongst older adults, yet by Multivariable Cox proportional hazard analysis this association disappeared and was replaced by sociodemographic differences. Inferior survival identified amongst older adults with ALL at HV and APs is contrary to many other epidemiologic studies of patients with HMs, which often describe superior survival outcomes at larger and academic facilities compared to smaller and community facilities [18, 20, 21, 22]. Two prior studies reported superior survival in adults ≥40 years with ALL treated at academic versus nonacademic centers based on NCDB data analysis collected from 1998 to 2012 [20, 22]. Conversely another publication found that older adults with ALL treated at academic centers versus nonacademic centers had worse survival [21]. Our results suggest that survival outcome differences between facilities may have occurred due to confounding sociodemographic differences. Currently there is a paucity of studies that focus on how ALL treatment and patient sociodemographic features vary by facility volume and type and how variation impacts outcomes. To our knowledge, data on adult ALL outcomes analyzed by facility volume has not been previously reported.

### 4.2 Sociodemographic Features

The impact of age in ALL is complex and important given divergent outcomes by age found in prior studies and in our study [5, 6, 7, 8]. We identified a significant difference associated with age on multivariable analysis, suggesting age is an important factor independently. We found patients ≥40 had a greater percentage of at least one major comorbidity compared to adults <40. Other ALL studies have found that patients with comorbidities had higher risk of death than those without comorbidities [21]. Despite this, increased comorbidities do not fully account for all differences in the older adult population compared to the younger cohort. This is supported in our multivariable analysis of patients ≥40, which found age, but not CCI, was associated with increased hazard of death. In addition to sociodemographic features, disease biology and treatment patterns vary by age cohort and thus influence outcomes [1, 5, 6, 7, 8, 11].

Baseline characteristic analysis found race was different by facility type but not volume, while ethnicity was different by facility volume but not type. For all facility volumes and types, nonHispanic White patients were the majority. A higher representation of nonHispanic White patients in the NCDB has been identified in prior studies and potentially reflects disparities in cancer care and research [23]. Similar to our study Khullar, Plascak, and Parikh found no difference in survival between white and Black patients, however unlike our findings, they identified decreased unadjusted hazard of death in Hispanic patients verse nonHispanic patients, whereas we identified increased hazard on adjusted analysis [21]. Hispanic Americans have been shown to have a higher incidence of ALL throughout life compared to White patients and they have a higher prevalence of Ph‐like ALL phenotype, a poor prognostic marker [24, 25]. Additionally Hispanic Americans may have a greater incidence of additional sociodemographic features that may influence poorer outcomes. Compared to white patients, those who identified as “Other” or Asian also had increased hazard of death, again possibly related to a greater incidence of additional sociodemographic features that negatively influence outcomes.

Insurance status has been found to influence outcomes in adult ALL. Krakora et al. found insured patients at time of diagnosis had longer progression‐free survival than patients without insurance [26]. Other NCDB studies of adult ALL patients have also found an association between having insurance and superior outcomes, specifically with respect to private insurance and variably medicare [18, 19, 20, 21, 22]. In our study, the majority of the patients ≥40 years of age had private or Medicare insurance versus none, and these insurance types were associated with the greatest reductions in hazard ratio among all of the variables investigated.

Our study also found living closer to the treatment facility was associated with decreased hazard of death. Living closer to the treatment facility was more common in patients treated at LV and CPs, while a greater percentage treated at HV and APs lived 10–100 or >100 miles from the treatment center. Similar to our study, other studies investigating ALL patients including those ≥40 years, identified that patients residing closer to treatment centers had a lower hazard of death [7, 21, 27]. Outcomes related to distance from clinic and area of residence is complex, likely confounded by other sociodemographic factors and impacted by disease‐specific nuances. ALL often requires specialized care available at specific facilities; however, patients can also develop emergent complications, potentially conferring benefit of closer treatment centers.

### 4.3 Treatment Patterns

Our study found more treatment was provided at HV and APs in older adults. Specifically, we found shorter TTT occurred at HV facilities. Analysis of treatment provided demonstrated HV and APs compared to LV and CP were more likely to provide all forms of treatment: immunotherapy, chemotherapy, radiation, and transplant. HV facilities were associated with the greatest delivery of anti‐neoplastic therapy. Our study found slightly older patients were more likely to receive care at LV and CPs, institutions which generally gave less treatment.

Multiagent chemotherapy in ALL is associated with improvement in survival, but to a different extent across age groups [11]. There has been an up‐trend in the receipt of chemotherapy in patients >65 years over the last decade, although patients with advanced age, compared to younger patients, are still less likely to receive anti‐neoplastic therapy. Additionally in today's treatment paradigm, utilization of better tolerated nonchemotherapy anti‐neoplastic therapy has become increasingly utilized in all age cohorts, particularly in patients with Ph(+) disease [10] and older patients [8, 9, 10, 11, 12, 13, 14, 15, 16]. Over the past decade there have been novel agents approved to treat historically poor risk groups, including older adults with relapsed/refractory ALL. Novel agents include the CD19 bispecific T cell engaging monoclonal antibody blinatumomab [16], anti‐CD22 antibody‐drug conjugate inotuzumab–ozogamicin [12], the antimetabolite nelarabine [9], and chimeric antigen receptor (CAR) T‐cell therapies [13, 14, 15]. The use of novel immunotherapy‐based therapeutics to‐date has been largely investigational, reserved for later stages of disease, and limited to specific facilities thus not reflected in NCDB data currently [6, 8]. Going forward it will be critical to observe the impact of these treatments as they continue to become incorporated as standard of care, with particular attention given to their impact stratified by facility volume and type, as well as by patient sociodemographics, to ensure maximally effective and equitable care delivery to all patient populations including older patients.

### 4.4 Limitations

The limitations of this study include its retrospective nature, which while informative, prohibited causal analysis. Our study is hypothesis generating, however, the relevance and robustness of our findings were limited by missing data including treatment facility type for patients <40 years, lack of ability to assess underlying disease risk/aggressiveness including but not limited to Ph status, lack of treatment details regarding specific agents administered and treatment after first line therapy, and the significant delay between release of NCDB files and changes in clinical practice. Analysis of patients ≥40 years was conducted primarily to fit within constraints of the NCDB database, not based on widely accepted age classification utilized in clinical practice (older adult ALL patients defined as >55–60 years). NCDB reports data from only the initial treatment center, thus key data was lost for patients that received subsequent therapy at a second facility, greatly limiting analysis and resultant conclusions. This limitation is evidenced by patients who were documented as receiving only palliative care, yet had mOS at all facility types that was greater than would be expected base on the natural history of disease, suggesting that a majority of these patients may have actually gone on to receive therapy at another facility that was not captured in the NCDB data files.

## 5 Conclusion

Treatment facility volume and type were not found to have a significant association with survival outcomes in older adults with ALL based on Multivariable Cox proportional hazard analysis, which demonstrated loss of facility volume or type as independent predictors while several sociodemographic factors were significant: age, race, ethnicity, insurance status, urban/rural residence, and distance from clinic. Future research is needed to fully characterize how sociodemographic factors influence adult ALL outcomes in real‐world clinical practice as treatment paradigms in ALL continue to evolve, particularly as related to treatment facility volume and type.

## Author Contributions

**Kaitlyn C. Dykes:** conceptualization (lead), data curation (equal), formal analysis (equal), investigation (lead), methodology (equal), project administration (equal), resources (lead), writing – original draft (lead), writing – review and editing (lead). **Jiling Chou:** formal analysis (lead), methodology (equal), writing – review and editing (equal). **Allison O. Taylor:** data curation (supporting), resources (equal), writing – review and editing (supporting). **Albert C. Shu:** resources (supporting), writing – review and editing (supporting). **Sarah E. Mudra:** resources (supporting), writing – review and editing (supporting). **Xiaoyang Ma:** methodology (supporting). **Jaeil Ahn:** formal analysis (supporting), methodology (supporting), supervision (supporting). **Catherine E. Lai:** conceptualization (lead), data curation (supporting), methodology (supporting), resources (supporting), supervision (lead), writing – review and editing (lead).

## Disclosure

This was an Institutional Review Board‐exempt study completed with publicly available data.

## Conflicts of Interest

The authors have no relevant conflicts of interest to disclose.

## Supporting information


**Table S1.** Kaplan–Meier (KM) overall survival (OS) analysis. The table provides *n*, events: treatments, mOS, 95% CI and *p*‐values. OS was censored by death or last follow‐up.
**Table S2.** Kaplan–Meier (KM) analysis for time to treatment (TTT) by facility volume and type for patients ≥40 years. TTT was censored by time defined as “treatment started, days from diagnosis.” Treatment included any systemic therapy, radiation, or surgery.
**Table S3.** Initial treatment received after diagnosis for patients ≥40, stratified by volume and facility type.

## Data Availability

The NCDB participant user files that support the findings of this study are openly available upon request at https://www.facs.org/quality‐programs/cancer‐programs/national‐cancer‐database/.
