# Supplementary material for: Survival Outcomes in Older Adult Acute Lymphoblastic Leukemia Patients Analyzed by Facility Volume and Type: A National Cancer Database Analysis
Source: Cancer Rep (Hoboken). 2024 Aug 8;7(8):e2162. [Article in Spanish] doi: 10.1002/cnr2.2162 (PMC11310087; doi:10.1002/cnr2.2162)
Supplement: Supplementary file 1 — Table S1. Kaplan–Meier (KM) overall survival (OS) analysis. The table provides n, events: treatments, mOS, 95% CI and p‐values. OS was censored by death or last follow‐up. Table S2. Kaplan–Meier (KM) analysis for time to treatment (TTT) by facility volume and type for patients ≥40 years. TTT was censored by time defined as “treatment started, days from diagnosis.” Treatment included any systemic therapy, radiation, or surgery. Table S3. Initial treatment received after diagnosis for patients ≥40, stratified by volume and facility type. [file CNR2-7-e2162-s001.docx]

**Supplement Tables & Figures:**

| **Age (years)** | **Center** | **n** | **Events: treatment** | **mOS (months)** | **95% CI** | **p** |
| --- | --- | --- | --- | --- | --- | --- |
| > 40 | CP | 4453 | 1070 | 69.2 | 65.0-72.6 | **0.00049** |
|  | AP | 8745 | 2729 | 61.2 | 58.5-63.4 |  |
|  | LV | 6394 | 1522 | 68.7 | 65.2-71.5 | **< 0.0001** |
|  | HV | 6804 | 2277 | 59.5 | 56.6-62.3 |  |
|  | LV CP | 3862 | 891 | 71.1 | 66.6-75.8 | **< 0.0001** |
|  | LV AP | 2532 | 631 | 64.7 | 61.2-70.7 |  |
|  | HV CP | 591 | 179 | 57.1 | 52.6-69.4 |  |
|  | HV AP | 6213 | 2098 | 59.6 | 56.6-62.7 |  |

**Supplement Table 1.** Kaplan-Meier (KM) overall survival (OS) analysis. The table provides n, events: treatments, mOS, 95% CI and P-values. OS was censored by death or last follow-up.

| **Age (years)** | **Center** | **n** | **Events: treatment** | **Median TTT (Days)** | **95% CI** | **p** |
| --- | --- | --- | --- | --- | --- | --- |
| > 40 | CP | 4405 | 3762 | 6 | 6-6 | **<0.0001** |
|  | AP | 9315 | 8797 | 5 | 5-5 |  |
|  | LV | 6329 | 5419 | 6 | 6-6 | **<0.0001** |
|  | HV | 7391 | 7140 | 5 | 5-5 |  |
|  | LV CP | 3770 | 3156 | 7 | 6-7 | **<0.0001** |
|  | LV AP | 2559 | 2263 | 6 | 5-6 |  |
|  | HV CP | 635 | 606 | 4 | 3-4 |  |
|  | HV AP | 6756 | 6534 | 5 | 5-5 |  |

**Supplement Table 2.** Kaplan-Meier (KM) analysis for time to treatment (TTT) by facility volume and type for patients > 40 years. TTT was censored by time defined as “treatment started, days from diagnosis.” Treatment included any systemic therapy, radiation, or surgery.

|  |  |  | **Center Volume (%)** | | | **Facility Type (%)** | | |
| --- | --- | --- | --- | --- | --- | --- | --- | --- |
|  | **Variable** | **Overall** | **Low ≤91%** | **High >91%** | **p** | **Community** | **Academic** | **p** |
| n |  | 14593 | 7004 | 7589 |  | 4885 | 9708 |  |
| Immunotherapy (%) | No | 13102 (90.5) | 6471 (93.4) | 6631 (87.8) | **<0.001** | 4490 (92.6) | 8612 (89.4) | **<0.001** |
|  | Yes | 1379 (9.5) | 458 (6.6) | 921 (2.2) |  | 361 (7.4) | 1018 (10.6) |  |
| Chemotherapy (%) | No | 1321 (9.5) | 1018 (15.9) | 303 (4.1) | **<0.001** | 728 (16.3) | 593 (6.3) | **<0.001** |
|  | Yes | 12517 (90.5) | 5394 (84.1) | 7123 (95.9) |  | 3739 (83.7) | 8778 (93.7) |  |
| Radiation (%) | No | 13041 (90.0) | 6577 (94.7) | 6464 (85.6) | **<0.001** | 4553 (93.8) | 8488 (88.0) | **<0.001** |
|  | Yes | 1453 (10.0) | 365 (5.3) | 1088 (14.4) |  | 299 (6.2) | 1154 (12.0) |  |
| Transplant (%) | No | 12312 (86.2) | 6503 (94.7) | 5809 (78.2) | **<0.001** | 4469 (93.0) | 7843 (82.7) | **<0.001** |
|  | Yes | 1977 (13.8) | 361 (5.3) | 1616 (21.8) |  | 337 (7.0) | 1640 (17.3) |  |
| Palliative (%) | No | 14040 (97.9) | 6814 (97.4) | 7226 (98.4) | **<0.001** | 4749 (97.4) | 9291 (98.2) | **0.002** |
|  | Yes | 294 (2.1) | 179 (2.6) | 115 (1.6) |  | 125 (2.6) | 169 (1.8) |  |

**Supplement Table 3.** Initial treatment received after diagnosis for patients > 40, stratified by volume and facility type.
